# *KRAS* G12S Positive Non‐Small Cell Lung Cancer Mimicking *KRAS* G12C Positivity on the AmoyDx PCR Panel: A Case Report

**DOI:** 10.1111/1759-7714.70361

**Published:** 2026-10-16

**Authors:** Tadashi Sakaguchi, Kentaro Ito, Miho Nishio, Yuta Suzuki, Akemi Iketani, Kengo Ushiro, Osamu Hataji

**Affiliations:** ^1^ Department of Respiratory Medicine Saiseikai Matsusaka Municipal Hospital Matsusaka Mie Japan; ^2^ Cancer Genomic Laboratory Saiseikai Matsusaka Municipal Hospital Matsusaka Mie Japan; ^3^ Department of Clinical Laboratory Saiseikai Matsusaka Municipal Hospital Matsusaka Mie Japan; ^4^ Department of Pathology Saiseikai Matsusaka Municipal Hospital Matsusaka Mie Japan

**Keywords:** AmoyDx pan lung cancer PCR panel, false‐positive, *KRAS* G12C, *KRAS* G12S, multi‐nucleotide variants

## Abstract

A false‐positive *KRAS* G12C on a multi‐PCR assay using the amplification refractory mutation system can potentially occur in cases harboring certain multi‐nucleotide variants other than *KRAS* G12C. A 62‐year‐old man with a large left upper lobe tumor was diagnosed with lung adenocarcinoma. A multi‐PCR assay by the AmoyDx Pan Lung Cancer PCR panel showed positivity for *KRAS* G12C and *KRAS* G12A/G12V/G12R/G13C. However, next‐generation sequencing revealed a *KRAS* variant of c.34_35delinsTC (p.G12S). *KRAS* G12S multi‐nucleotide variants may lead to false‐positive *KRAS* G12C results on the multi‐PCR assay.

## Graphical abstract

KRAS G12S (c.34_35delinsTC) can lead to false‐positive results for KRAS G12C on the AmoyDx PCR panel. Confirmation of the genetic mutation using alternative testing methods, such as NGS, is necessary to avoid inappropriate treatment.

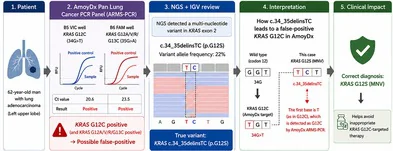

**Abbreviations**
AmoyDx: AmoyDx pan lung cancer PCR panel
ARMS: amplification refractory mutation system
IGV: Integrative Genomics Viewer
KRAS: Kirsten rat sarcoma virus
LCCP: lung cancer compact panel
MNV: multi‐nucleotide variant
NGS: next generation sequence
NSCLC: non‐small cell lung cancer
ODxTT: Oncomine Dx target test
OPA: oncomine precision assay
SNV: single‐nucleotide variant

## 1 Introduction

The Kirsten rat sarcoma virus (*KRAS*) mutations are detected in approximately 30% of Western lung adenocarcinomas and 10%–14% of Japanese lung adenocarcinomas [1, 2, 3]. KRAS G12C is an important therapeutic target, as sotorasib has demonstrated superior progression‐free survival and a favorable safety profile compared with docetaxel in previously treated advanced NSCLC [4, 5]. Accordingly, sotorasib monotherapy is recommended as a second‐line or later treatment in current guidelines [6, 7]. As the companion diagnostic tests, the Oncomine Dx Target Test (ODxTT), AmoyDx Pan Lung Cancer PCR Panel (AmoyDx), Lung Cancer Compact Panel (LCCP), Guardant360 assay, and therascreen KRAS PCR kit have been approved in Japan.

A previous report described a case in which a positive result for *KRAS* G12F, a rare *KRAS* mutation, may have led to a false‐positive result for *KRAS* G12C using the AmoyDx assay [8]. A false‐positive finding could result in the administration of inappropriate therapies, leading to disease progression, preventable toxicity, and substantial economic burden. Here, we report a case in which a positive result for *KRAS* G12S may also have led to a false‐positive *KRAS* G12C result on the AmoyDx assay.

## 2 Case Report

A 62‐year‐old man with a large tumor in his left upper lobe was referred to our hospital due to a suspicion of lung cancer. The forceps biopsies revealed lung adenocarcinoma with rich necrosis. He gave written consent and was enrolled in a prospective study about the comparison of the multi‐gene testing for NSCLC with necrosis‐rich tumors. He then underwent ODxTT and AmoyDx using his tissue samples, LCCP using his cytology sample, and Oncomine Precision Assay (OPA) using his plasma sample (the study was approved by the Clinical Research Ethics Committee of Matsusaka Municipal Hospital [approval number J‐322‐250 604‐2‐1. Approval date: June 9, 2023], and registered in the University Hospital Medical Information Network Clinical Trials Registry [UMIN000051503. Registry date: July 3, 2023]). As a result of multi‐gene testing, while the next generation sequence (NGS) results from LCCP and OPA showed positivity for *KRAS* G12S, ODxTT reported positivity for *KRAS* G12C. Furthermore, the multi‐PCR assay on AmoyDx reported positivity for *KRAS* G12C and *KRAS* G12A/G12V/G12R/G13C, but not for *KRAS* G12S (Figure 1). The therascreen KRAS PCR kit (Qiagen) confirmed the absence of the G12C mutation. The Integrative Genomics Viewer (IGV) data of NGS, including ODxTT, LCCP, and OPA, all concordantly showed positivity of the variant type of *KRAS* exon2 c.34_35delinsTC (p.G12S) (Figure 2); however, because the variant is not a reportable variant in the ODxTT assay, it was erroneously reported as *KRAS* G12C. These IGV data results from NGS suggested a false positive for *KRAS* G12C and *KRAS* G12A/G12V/G12R/G13C in AmoyDx. The patient was diagnosed with locally advanced lung adenocarcinoma and treated with Durvalumab after chemoradiotherapy.

**FIGURE 1:**
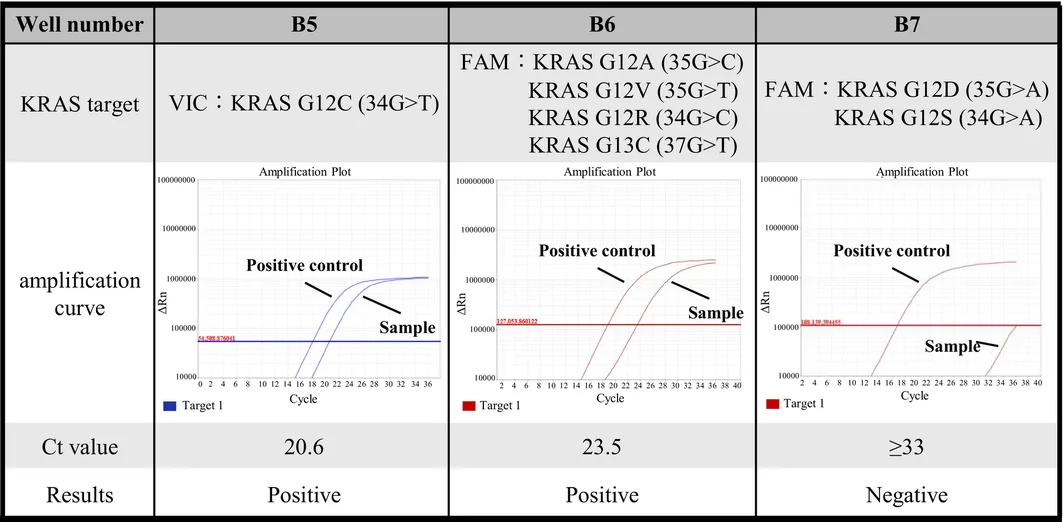
The result of *KRAS* mutations on AmoyDx. The result showed simultaneous positivity in both the B5 VIC well for detecting KRAS G12C and the B6 FAM well for detecting KRAS G12A/G12V/G12R/G13C. A, adenine; C, cytosine; Ct, cycle threshold; G, guanine; KRAS, Kirsten rat sarcoma virus; T, thymine.

**FIGURE 2:**
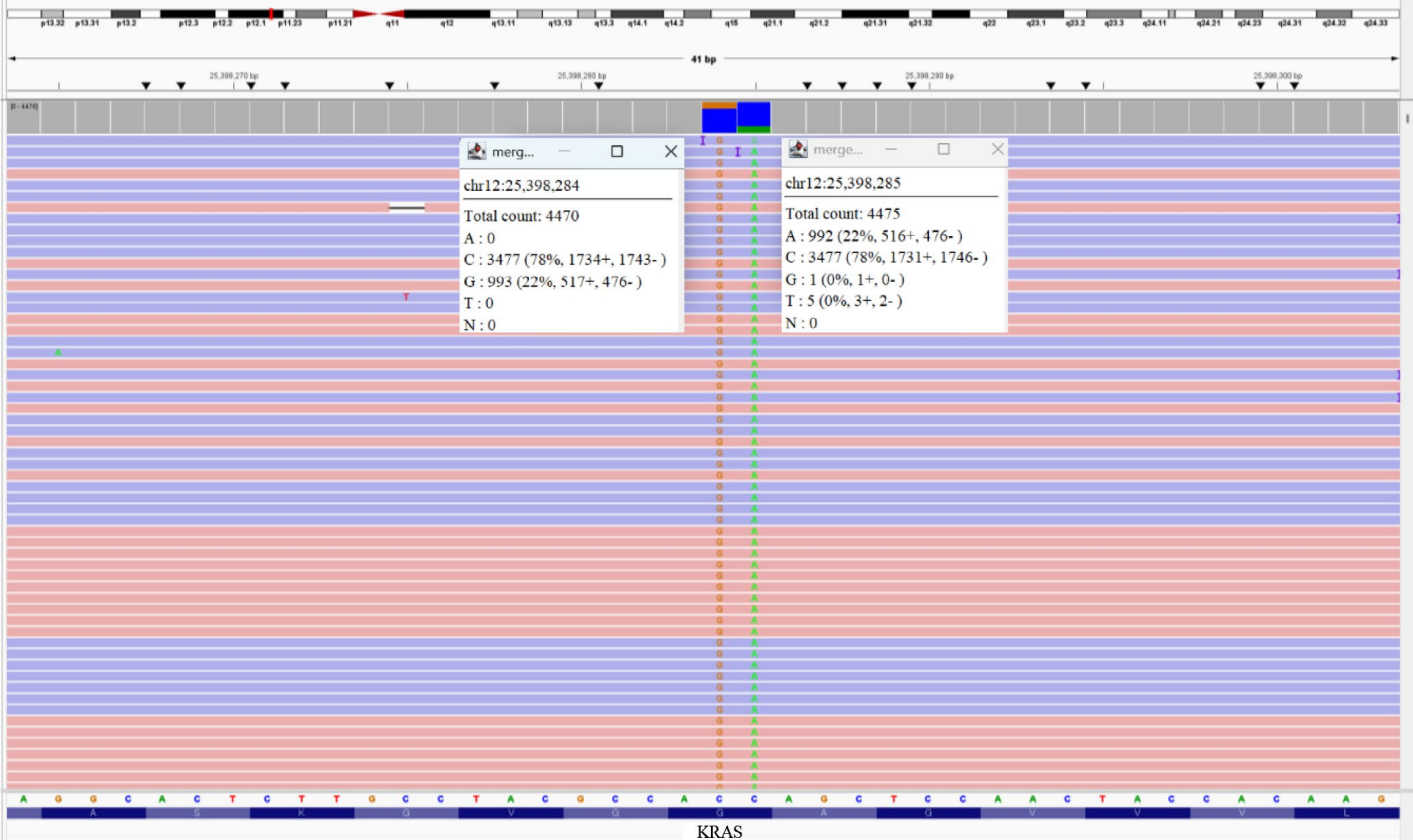
Integrative Genomics Viewer (IGV) data of *KRAS* on the Oncomine Precision Assay. The data showed positivity of a multi‐nucleotide variant in *KRAS* exon 2, c.34_35delinsTC (p.G12S), with a variant allele frequency of 22%. C, cytosine; KRAS, Kirsten rat sarcoma virus; T, thymine.

## 3 Discussion

In this report, we describe a case in which a positive result for *KRAS* G12S, a rare *KRAS* mutation, may have led to a false‐positive result for *KRAS* G12C in AmoyDx. The AmoyDx panel is based on the amplification refractory mutation system (ARMS), which uses 3′‐prime mismatches of the nucleotide changes [9]. In the detection of gene mutations in DNA using AmoyDx, when the region of the target gene mutation in the specimen DNA matches the base at the 3′ end of the primer, efficient amplification occurs, and the amplified product is detected by fluorescence using a hydrolysis probe. The list of *KRAS* G12X mutations and the reportable variants by AmoyDx are shown in Table 1 [10]. *KRAS* G12C is included as a companion diagnostic target of sotorasib, whereas *KRAS* G12A/V/R/D/S are included as reference information and are not classified as companion diagnostic targets. Among these mutations, only single‐nucleotide variants (SNVs) are included as detection targets in AmoyDx. The corresponding c.34G>T sequence is used to confirm the presence or absence of *KRAS* G12C in AmoyDx. However, *KRAS* G12S with multi‐nucleotide variants (MNVs) harbored in this case has also been found to exhibit c.34G>T, although it is not a reportable variant in AmoyDx. Furthermore, the variant has also been found to exhibit c.35G>C, which corresponds to the sequence used for the detection of *KRAS* G12A in AmoyDx. Therefore, the primers for *KRAS* G12C and *KRAS* G12A may have matched the sequence of *KRAS* G12S with MNVs, whereas the primer for detection of *KRAS* G12S with a SNV may not have matched this sequence. In the previously reported case of *KRAS* G12F‐positive NSCLC, mimicking *KRAS* G12C positivity is considered to have occurred in the same manner [8]. In addition, variants harboring c.34G>T, such as *KRAS* G12W and G12Y, may potentially exhibit the mimicking of *KRAS* G12C positivity. Details of the primer design have not been disclosed; therefore, these considerations remain hypothetical, and validation using samples harboring such actual MNVs is required.

**TABLE 1:** List of *KRAS* G12X mutations and reportable variants detected by AmoyDx.

*KRAS* G12 amino acid substitution	Exon	Base substitution			Variant type	COSMIC ID
		c.34G>	c.35G>	c.36T>		
G12C	2	**T**	G	T	c.34G>T	COSM516
	2	**T**	G	**C**	c.34_36delinsTGC	COSM513
G12V	2	G	**T**	T	c.35G>T	COSM520
	2	G	**T**	**C**	c.35_36delinsTC	COSM515
	2	G	**T**	**G**	c.35_36delinsTG	COSM4387521
G12D	2	G	**A**	T	c.35G>A	COSM521
G12A	2	G	**C**	T	c.35G>C	COSM522
	2	G	**C**	**A**	c.35_36delinsCA	COSM5413585
G12S	2	**A**	G	T	c.34G>A	COSM517
	2	**T**	**C**	T	c.34_35delinsTC	COSM6959652
G12R	2	**C**	G	T	c.34G>C	COSM518
	2	**A**	G	**A**	c.34_36delinsAGA	COSM249888
G12F	2	**T**	**T**	T	c.34_35delinsTT	COSM512
G12L	2	**C**	**T**	T	c.34_35delinsCT	COSM514
	2	**C**	**T**	**G**	c.34_36delinsCTG	COSM1716372
G12I	2	**A**	**T**	T	c.34_35delinsAT	COSM34144
G12W	2	**T**	G	**G**	c.34_36delinsTGG	COSM36281
G12E	2	G	**A**	**A**	c.35_36delinsAA	COSM519
	2	G	**A**	**G**	c.35_36delinsAG	COSM30566
G12Y	2	**T**	**A**	T	c.34_35delinsTA	COSM25081
G12N	2	**A**	**A**	T	c.34_35delinsAA	COSM13643
G12H	2	**C**	**A**	T	c.34_35delinsCA	COSM7335307

*Note:* Bold indicates a substituted base, Gray indicates a reportable variant by AmoyDx.

Abbreviations: A, adenine; AmoyDx, AmoyDx Pan Lung Cancer PCR Panel; C, cytosine; COSMIC, the catalogue of somatic mutations in cancer; G, guanine; KRAS, Kirsten rat sarcoma virus; T, thymine.

As demonstrated in the present case, IGV review of NGS data can be highly informative when a false‐positive KRAS G12C result from AmoyDx is considered. However, reimbursement restrictions in Japan limit the use of additional multigene assays for confirmatory testing in clinical practice. Broader access to orthogonal confirmatory testing, including NGS‐based assays, would be desirable in cases of considered false‐positive results.

To our knowledge, this is the first case in which a positive result for *KRAS* G12S may have led to a false‐positive result for *KRAS* G12C in AmoyDx. In AmoyDx, simultaneous positivity in both the B5 VIC well for detecting *KRAS* G12C and the B6 FAM well for detecting *KRAS* G12A/G12V/G12R/G13C may indicate a co‐mutation. The possibility, however, that *KRAS* G12F or G12S could result in mimicking *KRAS* G12C positivity should also be considered. Furthermore, in theory, a similar phenomenon may occur with MNVs harboring c.34G>T, such as KRAS G12W and G12Y. In such situations, confirmation of the genetic mutation using alternative testing methods, such as NGS, is necessary to avoid inappropriate treatment.

## Author Contributions

**Kentaro Ito:** conceptualization, methodology, supervision, writing – review and editing. **Tadashi Sakaguchi:** conceptualization, writing – original draft, methodology, resources, visualization. **Osamu Hataji:** funding acquisition, supervision, writing – review and editing. **Kengo Ushiro:** resources, writing – review and editing. **Akemi Iketani:** resources, writing – review and editing, conceptualization, methodology. **Miho Nishio:** conceptualization, methodology, resources, visualization, writing – review and editing. **Yuta Suzuki:** resources, writing – review and editing.

## Funding

The authors have nothing to report.

## Ethics Statement

Written informed consent was obtained from the patient for publication of this case report and any accompanying images.

## Conflicts of Interest

The authors declare no conflicts of interest.

## Data Availability

All data in this case report are included in this article. Further enquiries can be directed to the corresponding author.

## References

[1] I. A. Prior , F. E. Hood , and J. L. Hartley , “The Frequency of Ras Mutations in Cancer,” Cancer Research 80, no. 14 (2020): 2969–2974. 10.1158/0008-5472.CAN-19-3682 PMC7367715 32209560

[2] M. Saito , K. Shiraishi , H. Kunitoh , S. Takenoshita , J. Yokota , and T. Kohno , “Gene Aberrations for Precision Medicine Against Lung Adenocarcinoma,” Cancer Science 107, no. 6 (2016): 713–720. 10.1111/cas.12941 PMC4968599 27027665

[3] Y. Tamiya , S. Matsumoto , Y. Zenke , et al., “Large‐Scale Clinico‐Genomic Profile of Non‐Small Cell Lung Cancer With KRAS G12C: Results From LC‐SCRUM‐Asia Study,” Lung Cancer 176 (2023): 103–111. 10.1016/j.lungcan.2022.12.019 36634571

[4] J. Canon , K. Rex , A. Y. Saiki , et al., “The Clinical KRAS(G12C) Inhibitor AMG 510 Drives Anti‐Tumour Immunity,” Nature 575, no. 7781 (2019): 217–223. 10.1038/s41586-019-1694-1 31666701

[5] A. J. de Langen , M. L. Johnson , J. Mazieres , et al., “Sotorasib Versus Docetaxel for Previously Treated Non‐Small‐Cell Lung Cancer With KRAS(G12C) Mutation: A Randomised, Open‐Label, Phase 3 Trial,” Lancet 401, no. 10378 (2023): 733–746. 10.1016/S0140-6736(23)00221-0 36764316

[6] G. J. Riely , D. E. Wood , D. S. Ettinger , et al., “Non‐Small Cell Lung Cancer, Version 4.2024, NCCN Clinical Practice Guidelines in Oncology,” Journal of the National Comprehensive Cancer Network 22, no. 4 (2024): 249–274. 10.6004/jnccn.2204.0023 38754467

[7] I. A. Jaiyesimi , N. B. Leighl , N. Ismaila , et al., “Therapy for Stage IV Non‐Small Cell Lung Cancer With Driver Alterations: ASCO Living Guideline, Version 2023.3,” Journal of Clinical Oncology 42, no. 11 (2024): e1–e22. 10.1200/JCO.23.02744 38417091

[8] I. Oi , T. Ito , Z. Saito , et al., “Kristen Rat Sarcoma Virus (KRAS) G12F‐Positive Non‐Small Cell Lung Cancer Mimicking KRAS G12C Positivity: A Case Report,” Thorac Cancer 15, no. 3 (2024): 271–273. 10.1111/1759-7714.15171 PMC10803219 38010032

[9] C. R. Newton , A. Graham , L. E. Heptinstall , et al., “Analysis of Any Point Mutation in DNA. The Amplification Refractory Mutation System (ARMS),” Nucleic Acids Research 17 (1989): 2503–2516. 10.1093/nar/17.7.2503 PMC317639 2785681

[10] J. G. Tate , S. Bamford , H. C. Jubb , et al., “COSMIC: The Catalogue of Somatic Mutations in Cancer,” Nucleic Acids Research 47, no. D1 (2019): D941–D947. 10.1093/nar/gky1015 PMC6323903 30371878

